# Directly ambient pressure dried robust bridged silsesquioxane and methylsiloxane aerogels: effects of precursors and solvents†

**DOI:** 10.1039/c8ra08646j

**Published:** 2019-03-15

**Authors:** Dangjia Chen, Hongyi Gao, Panpan Liu, Pei Huang, Xiubing Huang

**Affiliations:** Beijing Advanced Innovation Center for Materials Genome Engineering, Beijing Key Laboratory of Function Materials for Molecule & Structure Construction, School of Materials Science and Engineering, University of Science and Technology Beijing No. 30, Xueyuan Road, Haidian District Beijing 100083 PR China hygao2009@163.com +86-10-62333765

## Abstract

Robust low-cost silica based aerogels can be obtained by choosing appropriate silane precursors and chemical conditions. In this paper, we synthesized two kinds of bridged siloxane precursors, bridged silsesquioxane (BSQ) from (3-aminopropyl)-triethoxysilane (APTES) and *m*-phthalaldehyde (MPA), and bridged methylsiloxane (BMSQ) from (3-aminopropyl)-diethoxymethylsilane (APDEMS) and *m*-phthalaldehyde (MPA) to prepare robust aerogels. Methanol and ethanol were used individually as solvents in the experiment and all the products were dried directly at ambient pressure without any solvent exchange process. All the products show low densities (about 0.15 g cm^−3^) and large porosities (larger than 80%). The influence of the precursor and solvent was investigated. The BSQ aerogels have larger specific surface areas, smaller pore sizes and more stable mechanical performances. Aerogels prepared using methanol as the solvent gel faster and have larger pore sizes. The solvent has greater impacts on the BSQ aerogels, the BSQ aerogels prepared using ethanol as the solvent can withstand 60% deformation in repeated compression tests, exhibiting good mechanical performance.

## Abstract 2

Aerogels prepared with different precursors and solvents using a facile procedure.

## Graphical abstract

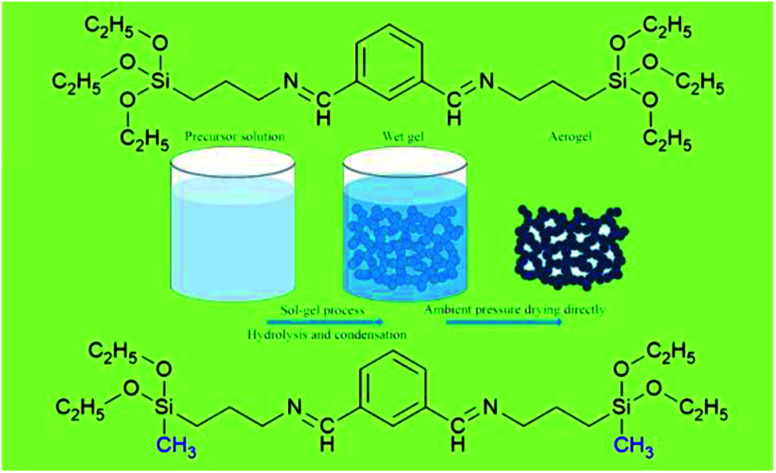

## Introduction

Aerogels are porous materials with fascinating properties such as high specific surface area and porosity, low density and low thermal conductivity.^1^ They have been applied in various fields ranging from thermal insulators and absorbents to Cherenkov radiation detectors.^2–4^ However, they have some drawbacks, such as their fragility and the laborious drying methods, which restrict their large-scale commercial applications. In order to enhance their robustness and reduce the cost of the aerogels, researchers have made great attempts to gain deep insights into the sol–gel techniques and search for facile drying methods.

Up to now, various kinds of strategies have been developed to enhance the robustness of aerogels.^5,6^ Many robust silica-based aerogels were obtained by incorporating the silica networks with reinforcements, such as organic polymers or fibers.^7–9^ Among the different reinforcing methods, it is effective to use bridged silsesquioxanes (BSQ) as precursors to prepare flexible aerogels.^10–13^ In these reports, the organic groups were directly and covalently bonded between the Si atoms in the precursor molecules, which gifted the aerogels with large degrees of freedom in designing the structures and modulating their properties. There were many reports about flexible aerogels prepared by BSQ *via* a facile procedure, but only a few aerogels were prepared from the analogues of BSQ, bridged methylsiloxanes (BMSQ).^14,15^ The BMSQ have a methyl group and two alkoxy groups instead of three alkoxy groups attaching to the silicon atom in their corresponding bridged silsesquioxanes. Besides the benefits from the strengthened bridge organic segment, the decreased cross-linking density of siloxane bonds and the pendent methyl groups in BMSQ may also contribute the resilient behaviour against compression,^14^ so it is a promising strategy to prepare robust hydrophobic aerogels using the BMSQ precursors.

The performance of aerogels can be regulated by the chemical conditions, such as pH value, solvents and start monomers.^16–21^ By controlling the hydrolysis and condensation reaction, the growth of particle and the properties of aerogels can be tailored. Solvents can influence the nucleation and growth of aerogels in the sol–gel process, and thereby the structures of final materials, for the different viscosity, polarity, and protic or non-protic behavior.^21^ Moreover, the drying techniques of aerogels can be simplified by choosing the proper solvents. Researchers have proved that the shrinkage, density, porosity, pore volume and some other properties of aerogels can be regulated by solvents.^20–23^ However, the influences of solvents on the new emergences, BSQ and BMSQ aerogels are rarely discussed. By changing the solvents, it is possible to modulate the properties of aerogels prepared by bridged siloxanes.

**Scheme 1:**
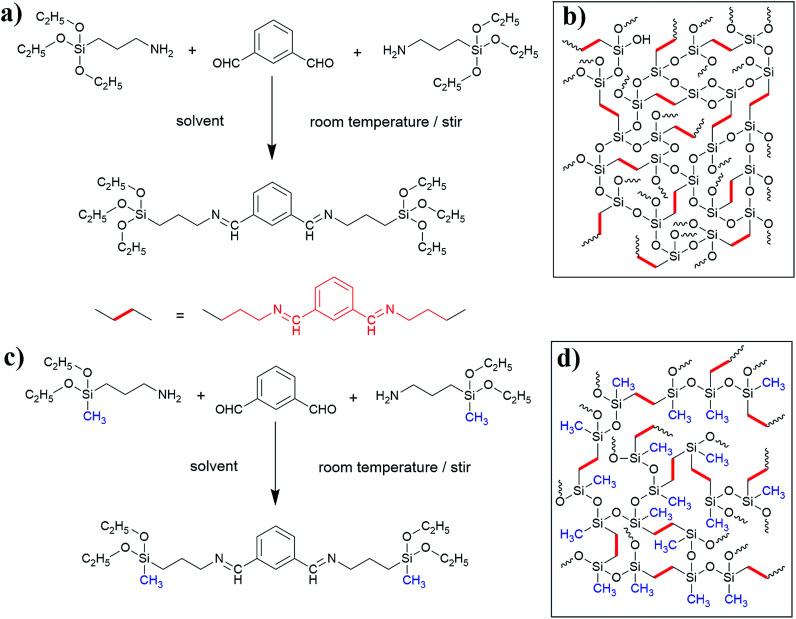
The synthesis scheme of the precursors and illustrated chemical structure of aerogels, (a) the synthesis scheme of BSQ precursor, (b) the illustrated chemical structure of BSQ aerogel, (c) the synthesis scheme of BMSQ precursor and (d) the illustrated chemical structure of BMSQ aerogel.

To prepare robust aerogels with low costs, two kinds of new precursors synthesized from (3-aminopropyl)-triethoxysilane (APTES)/*m*-phthalaldehyde (MPA) and (3-aminopropyl)-diethoxymethylsilane (APDEMS)/*m*-phthalaldehyde (MPA) are used to prepare the BSQ aerogels and the BMSQ aerogels in this paper. Methanol and ethanol are used individually as solvent. All the products are prepared *via* a very facile procedure. The synthesis process and the proposed molecular structure of the obtained aerogels are illustrated in Scheme 1. In the experiment, all the aerogels are directly dried at ambient pressure without any solvent exchange. Ambient pressure drying (APD) method has been regarded as a promising drying technique to product aerogels with more efficiency and safety. Aerogels need robust networks to endure the capillary force during drying. The aerogels in this work are all flexible and robust enough to dry at ambient pressure, all the aerogels can withstand a deformation of 60% strain at compression and the Young's moduli range from 0.12 to 1.14 MPa. The BMSQ aerogels are more capable to resist deformation due to their thicker networks. The solvents have greater impacts on the BSQ aerogels, BSQ aerogels prepared using ethanol as solvent have larger specific surface areas and more stable mechanical performance at repeating compression.

## Experimental

### Materials and methods

MPA, APTES and APDEMS were bought from Adamas Reagent. Analytically pure ethanol and methanol were obtained from Sinopharm Chemical Reagent. All the reagents were used without further purification.

**Scheme 2:**
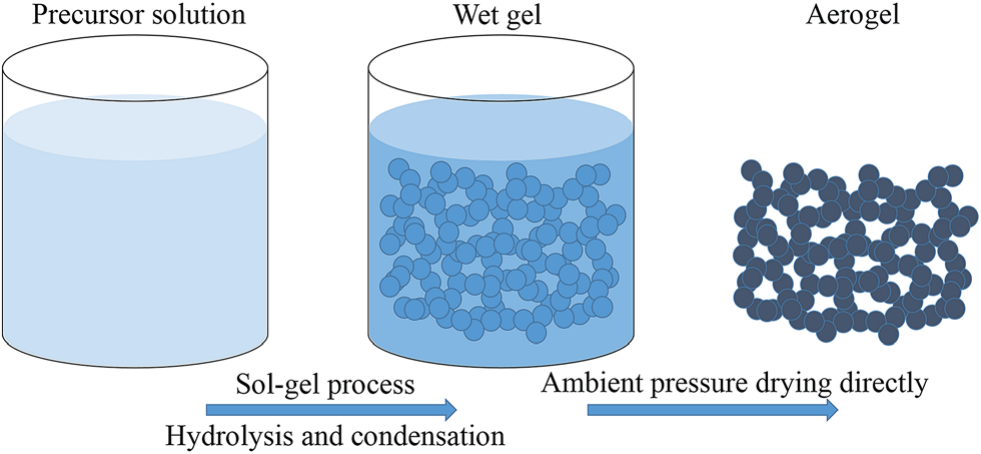
Schematic description for the facile procedure of aerogels.

Aerogels were synthesized using a simple one-pot method. The detailed formulas of all the products are list in Table S1.† The products are denoted as TME, TMM, DME and DMM according to the different precursors and solvents. The initial ‘T’ or ‘D’ means that the precursor of aerogel is APTES or APDEMS, the last ‘E’ or ‘M’ means the solvent is ethanol or methanol, ‘M’ in the middle means the precursor MPA. All the aerogels were prepared in the same procedure except for the difference in precursor and solvent. In a representative procedure, APTES (1 mL, 4.27 mmol) and MPA (0.28 g, 2.13 mmol) were added into methanol (7 mL) with a fixed molar ratio of 2 : 1, then the mixture was sonicated to form a homogeneous solution for 10 minutes, after then, 0.5 mL water was added into the above homogeneous solution. The obtained solution was then sonicated for another 10 minutes before transferred into a 20 mL syringe mold. Finally the sealed cylindrical mold was put into an oven for gelation at 60 °C. After sufficient gelation for 24 hours, the gel was ambient pressure dried directly at 20 °C for 24 hours and then at 80 °C for another 12 hours to remove the residual substance. After then, the final aerogel was obtained. The preparation procedure is illustrated as Scheme 2.

### Characterization

Fourier transform infrared (FT-IR) spectra were recorded on a NICOLET 6700 instrument to study the chemical bonding of the aerogels. Liquid-state ^13^C nuclear magnetic resonance (NMR) spectra were obtained on a Bruker 400M instrument with chloroform-d as solvent. Solid-state ^13^C and ^29^Si NMR study was carried out on a Bruker Avance III 400. The microstructural observation of the aerogels was performed using a Zeiss Supra 55 field emission scanning electron microscope (SEM). The densities of aerogels were calculated from the mass-to-volume ratios of each individual aerogel. The mass of an aerogel was obtained by an analytical balance with readability of 0.0001 g (Analytical Balance, Mettler Toledo) and the dimensions of an aerogel were measured by a vernier caliper. The shrinkage was obtained from the diameter of the aerogel monolith and the diameter of the 20 mL syringe mold. Nitrogen sorption isotherms were obtained using ASAP2460 (Micromeritics Instrument Corporation), the specific surface area was calculated using the BET (Brunauer–Emmett–Teller) method and the pore size distribution was obtained from the adsorption branch using the BJH (Barrett–Joyner–Halenda) method. Mercury intrusion porosimetry (MIP) analysis was conducted on AutoPore IV 9500 to determine the specific surface area, pore size distribution and porosity of aerogels. The mechanical properties were investigated on an Instron 4505 test stand using the repeated compression test with a compression rate of 10 mm min^−1^. The thermal conductivity was measured using a Hot Disk (TPS 2500) instrument contact angle measurements were carried out to determine the hydrophobicity of the aerogels on a JC2000 Contact Angle Measuring Device, POWEREACH.

## Results and discussion

**Fig. 1:**
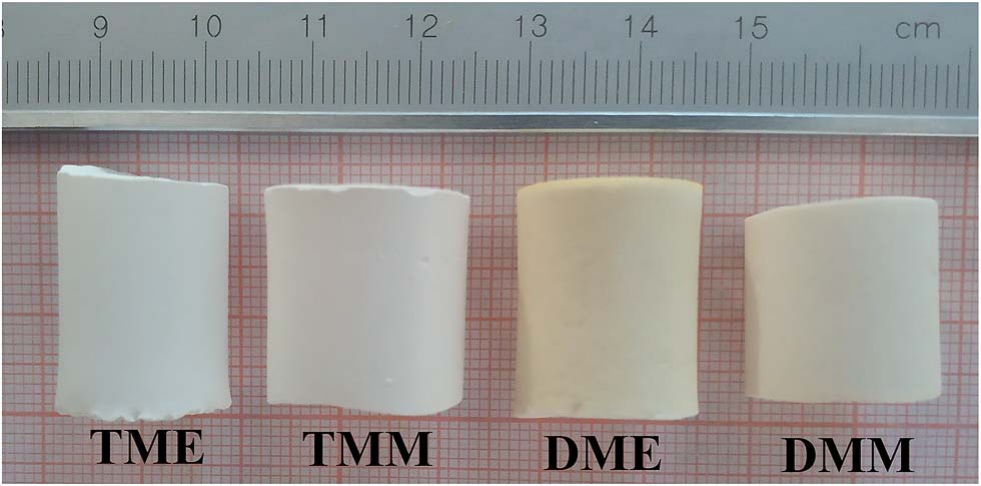
The optical images of the aerogels.

All the prepared aerogels are cylindrical monoliths, just as shown in Fig. 1. The physical properties of the aerogels are listed in Table 1. TME suffers the largest shrinkage since it has the smallest average pore size, which results in greatest capillary force during the drying process. Because of the largest shrinkage, TME has the lowest porosity of all the products. DME and DMM do not show much difference in the properties, indicating that the solvents have small influence on the BMSQ aerogels. The thermal conductivity of the aerogels is low due to the typical porous structure,^10,11^ the BMSQ aerogels have larger thermal conductivity than BSQ aerogels for the thicker skeleton and larger pore size,^24^ which can be seen in the SEM images in the following content. It should be noted that all the aerogels are directly dried at ambient pressure without any additional solvent exchange. This can be explained as: (1) the pores in aerogels are mainly macropores, indicating that the aerogels would not suffer great capillary force during the drying process; (2) ethanol and methanol are both volatile, and they would not cause very strong capillary force; (3) amino could easily react with aldehyde and the synthetic yield is high,^25^ after sufficient gelation and aging, there would not be much residual substance remaining in the aerogel pores to affect drying; (4) the skeletons of the aerogels are robust enough to withstand the capillary force during the drying process. Some other solvents were also used in this experiment (Fig. S1†), the BSQ products prepared using alcohols and *N*,*N*-dimethylformamide as solvent are white cylindrical monoliths, and despite of the obvious shrinkage, the BSQ products can keep their shapes after drying directly at ambient pressure. However, the skeleton of BMSQ products prepared using the solvents collapsed badly after drying, indicating that high boiling point solvents are not suitable for the directly ambient pressure drying technique in this experiment. The products prepared using acetone as solvent were deep red translucent monoliths before drying and they fragmented after drying directly at ambient pressure, it means that solvents have great effects on the structure of the aerogels.

**Table 1:** The physical properties of the obtained aerogels^a^

Sample	*ρ* (g cm^−3^)	*η* (%)	SSA^b^ (m^2^ g^−1^)	SSA^c^ (m^2^ g^−1^)	Pore size^d^ (μm)	*P* e (%)	*E* (MPa)	*κ* (W (m K)^−1^)
TME	0.149 ± 0.005	16.8 ± 0.6	68.68 ± 0.12	81.77 ± 0.15	0.35 ± 0.06	81.6 ± 0.8	0.12 ± 0.01	0.042 ± 0.0009
TMM	0.112 ± 0.009	7.9 ± 0.9	12.57 ± 0.03	2.31 ± 0.08	10.89 ± 0.02	89.7 ± 0.1	0.16 ± 0.01	0.047 ± 0.0010
DME	0.168 ± 0.006	15.3 ± 0.6	2.68 ± 0.02	1.79 ± 0.05	13.71 ± 0.02	86.5 ± 0.2	1.12 ± 0.02	0.051 ± 0.0009
DMM	0.161 ± 0.008	15.8 ± 0.7	1.76 ± 0.02	1.90 ± 0.03	18.68 ± 0.02	88.4 ± 0.2	1.14 ± 0.02	0.058 ± 0.0010

a *ρ*: density; *η*: the shrinkage of aerogel; *P*: porosity; *E*: Young's modulus; *κ*: thermal conductivity.

b SSA is the BET specific surface area.

c SSA is the specific surface area determined by MIP.

d The porosity is identified by MIP.

e The pore size is identified by MIP; *E* denotes the Young's modulus.

### Chemical characterization

**Fig. 2:**
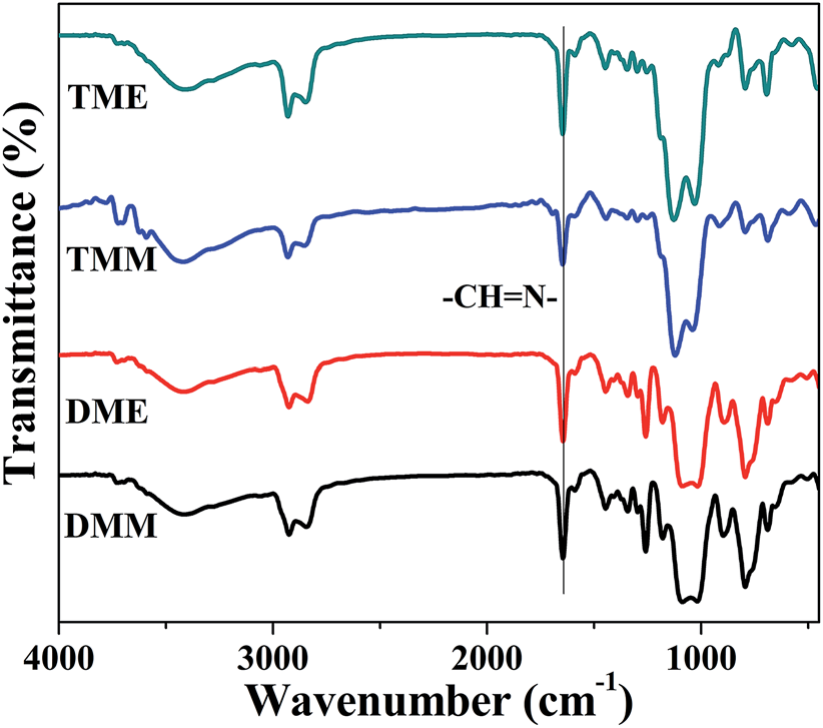
The FT-IR spectra of the obtained aerogels.

The Schiff base groups synthesized from amino and aldehyde can be easily distinguished from the FT-IR spectra of the aerogels (Fig. 2), the Schiff base (–CH=N–) stretch appears at 1643 cm^−1^, and the carbonyl group from MPA (Fig. S2†) is absent in the aerogel samples, which means that the reaction of amino groups and aldehyde groups was almost completed. The peaks at 2980 and 2960 cm^−1^ of all the aerogels are ascribed to the C–H bonds from APDEMS or APTES, which contributed to the hydrophobicity of the aerogels. The BMSQ aerogels are hydrophobic (Fig. S3†), but the BSQ aerogels can absorb water gradually (Fig. S4†). It shows that the pendent methyl groups in the BMSQ aerogels is in favour of increasing the hydrophobicity.

**Fig. 3:**
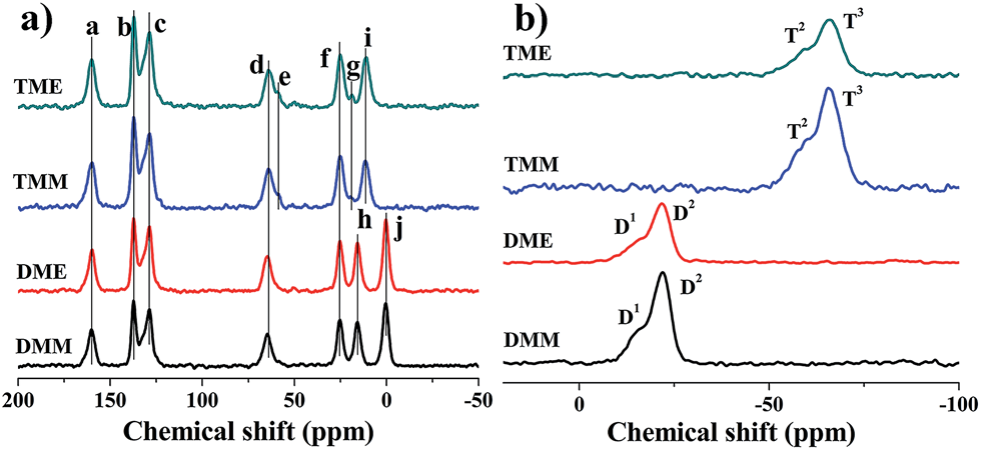
The NMR spectra of the aerogels, (a) ^13^C NMR spectra, (b) ^29^Si NMR spectra.

**Scheme 3:**
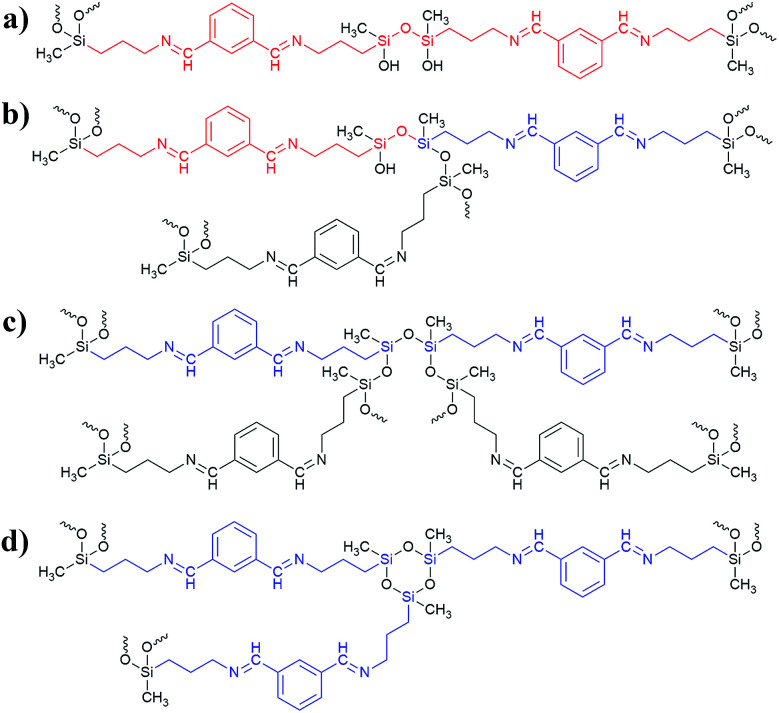
The possible linkage of the Si atoms in BMSQ aerogels; the linear segment attached to D^1^ Si atoms are marked in red, the linear segment attached to D^2^ Si atoms are marked in blue.

Further investigations of the chemical compositions of the aerogels are conducted by ^13^C and ^29^Si solid-state NMR spectroscopy (Fig. 3). As shown in Fig. 3a, all the peaks are labeled from ‘a’ to ‘j’. Compared with the ^13^C NMR spectra of the start monomers (Fig. S5†), the peak ‘a’ is from the carbon atoms of the Schiff bases, the peaks ‘b’ and ‘c’ are from the carbon atoms in the benzene ring. The peaks ‘d’, ‘f’ and ‘i’ (or ‘h’) are carbon atoms from the alkyl chain. The typical peak ‘j’ in DME and DMM is derived from the methyl groups in APDEMS. There are two more peaks ‘e’ and ‘g’ in TME and TMM, which indicate the incomplete hydrolysis of the precursors. Aerogels prepared from the same precursor show the same peaks. Although solvents affect the hydrolysis and condensation of aerogels in the sol–gel process, the NMR results suggest that solvents have almost no effects on the final chemical compositions of aerogels. From the result of ^29^Si NMR spectra (Fig. 3b), the condensations of all the aerogels are incomplete. The peaks in Fig. 3b are denoted as D^*i*^ or T^*i*^, where D^*i*^ refers to the peaks derived from the R′R′′ = Si(OSi)_*i*_(OR)_2−*i*_ species (R = H or Et, *i* = 1, 2), T^*i*^ refers to the peaks derived from the R′–Si(OSi)_*i*_(OR)_3−*i*_ species (R = H or Et, *i* = 2, 3) (R′ and R′′ denote the alkyl groups). For TME and TMM, the ^29^Si NMR spectra are almost the same, there are high peaks T^3^ in the spectra and distinguishable peaks T^2^ located beside T^3^. The spectra of DME and DMM resemble each other, besides the high peaks D^2^, there are obvious peaks D^1^. The results indicate that the condensations of the aerogels are not complete. For the high peak D^1^, we can infer that the cross-link densities of DME and DMM are low and there are long linear segments in DME and DMM (Scheme 3).

### Morphology and pore structure

**Fig. 4:**
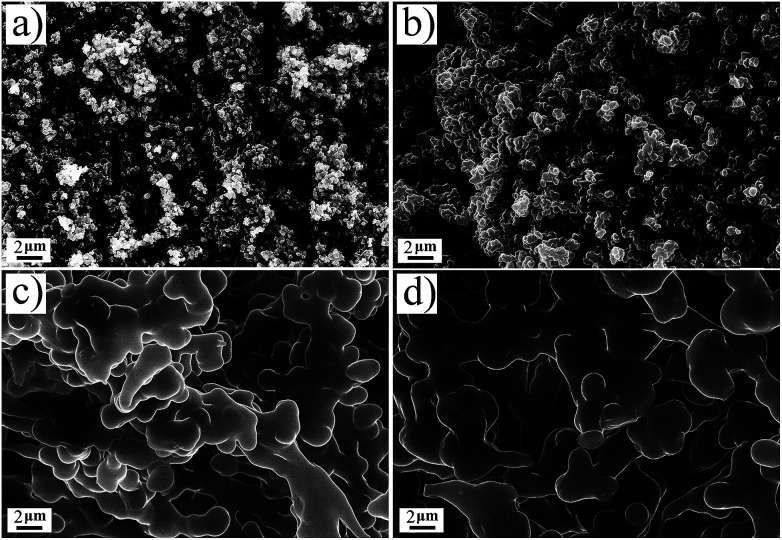
The SEM images of the aerogels, (a) TME, (b) TMM, (c) DME and (d) DMM.

Fig. 4 shows the SEM images of aerogels, it is obvious that TME has the smallest particle size. The particle size of TMM is larger than that of TME, suggesting that methanol is more favourable for the particle growth of BSQ aerogel than ethanol. DME and DMM have the similar particle size, which means that it is the precursor but not the solvent dominating the size of particles for the BMSQ aerogel. It is apparent that the particle size of the BMSQ aerogel is larger than that of BSQ aerogel, which is resulted from the slow hydrolysis and condensation rate of the BMSQ precursor, for the slow hydrolysis and condensation rate can lead to large particles.^26–30^ SEM images with higher magnification are shown in Fig. S6† to provide more details of the micromorphology of TME and TMM. The average particle size of TME is about 150 nm, which is the smallest of all. The hydrolysis and condensation rate is demonstrated by the gelation time for the aerogels, as shown in Fig. S7.† TMM has the shortest gelation time, while DME is still solution after 10 h. The result shows that the hydrolysis and condensation rate of the BMSQ precursor is much slower. According to Fig. S7,† aerogels gel faster in the methanol, which agrees with the previous reports.^20,28–33^ Although BSQ precursor shows faster hydrolysis and condensation rate in methanol, BSQ aerogels with larger particle size were obtained. The probable reason can be explained that methanol with lower viscosity has fewer tendencies to retard the particle aggregation of the BSQ aerogels, so the particles grow larger in methanol.

**Fig. 5:**
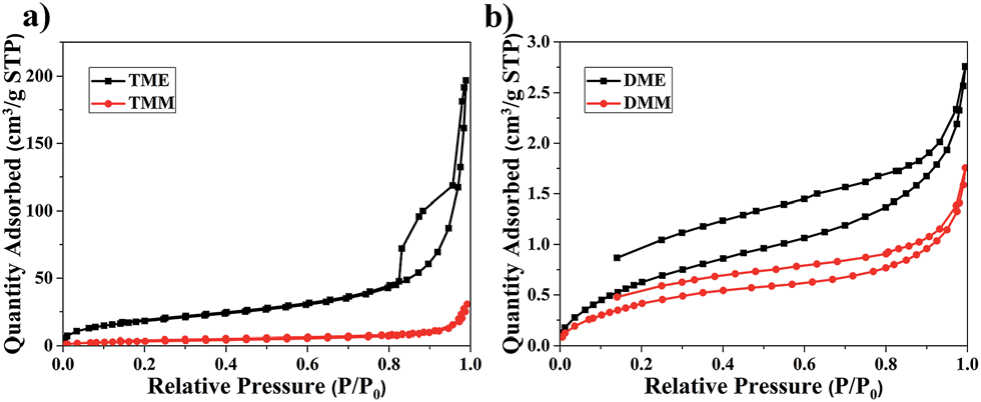
The nitrogen adsorption and desorption isotherms of the aerogels, (a) TME and TMM, (b) DME and DMM.

The pore structures of the aerogels were characterized by the nitrogen sorption method. The nitrogen sorption isotherms of the aerogels are shown in Fig. 5 and the pore size distributions are plotted in Fig. S8.† The N_2_ adsorption and desorption isotherm of TME appears between typical Type II plots and IV plots in IUPAC classification, the obvious hysteresis loop is frequently associated with mesopores. The isotherm of TMM appears typical Type II plots, indicating that there are almost macropores in TMM. The isotherms of DME and DMM appear between Type II and IV plots, but the absorbance is very low. This may be due to the low specific surface areas and the dominant macropores of the aerogels. The isotherms are not close at low relative pressure, it probably comes from the partial polymer structure of DME and DMM.^34^ The BET specific surface areas of the aerogels are low due to the large size particles and macropores in aerogels. From Table 1, the specific surface areas of the BMSQ aerogels are significantly smaller than those of the BSQ aerogels. Because of the slower hydrolysis and condensation rate, BMSQ aerogels with larger particle sizes and lower specific surface areas were obtained. Aerogels prepared using methanol as solvent have smaller specific surface areas, this can be explained from Fig. 4 that the aerogels prepared in methanol have larger particle size, as the particles grow larger, the specific surface areas are reduced.

**Fig. 6:**
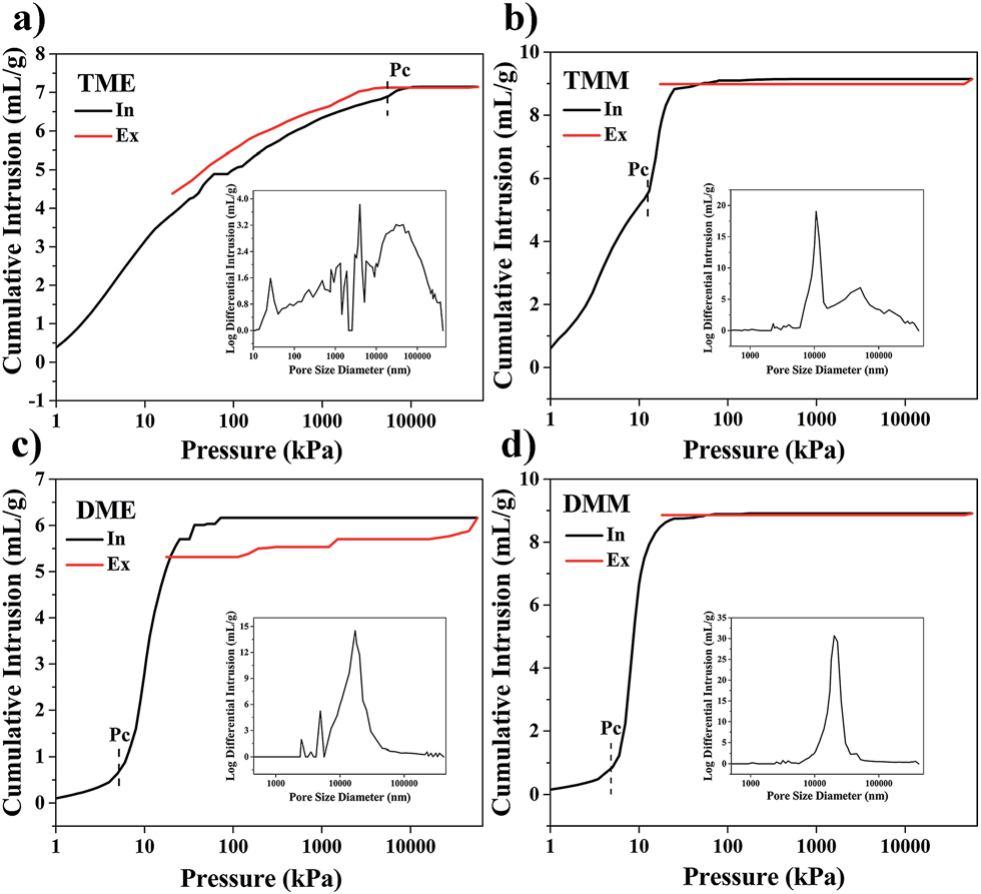
The mercury intrusion porosimetry (MIP) analysis results of aerogels, (a) TME, (b) TMM, (c) DME and (d) DMM.

The pore structures were further investigated by mercury intrusion porosimetry (MIP) analysis. As shown in Fig. 6, there are obvious differences between the MIP curves of TME and TMM, but the MIP curves of DME and DMM resemble each other, indicating that the effects of solvents on the pore structures of BSQ aerogels are greater than those of the BMSQ aerogels. The porosities of the aerogels and the specific surface areas are listed in Table 1. According to the reports from R. Pirard,^35,36^ aerogels are compacted but not intruded by the mercury when submitted to a pressure of mercury, so there exists a pressure *P*_c_ at which mercury begins to intrude the aerogels instead of crushing the network. There is an abrupt increase at *P*_c_ in the mercury intrusion curve for each aerogel. The pressure *P*_c_ was labelled in each curve, the *P*_c_ of the BMSQ aerogels is much smaller. According to the Washburn equation,^37^ the larger *P*_c_ means the smaller pore radius, therefore the pore diameters of the BMSQ aerogels are larger. The *P*_c_ of DME and DMM are almost equal, while the *P*_c_ of TME is much larger than that of TMM, suggesting the pore sizes of the BSQ aerogels are affected more easily by solvents than those of the BMSQ aerogels and it is the precursor but not the solvent dominating the pore sizes of BMSQ aerogels. The pore size distribution curves of the aerogels are inserted in Fig. 6, the pore size distribution of aerogels prepared using methanol as solvent is relatively centralized. The pore sizes of DME distribute widely ranging from 2 to 45 μm. For TME, there are many irregular peaks in the pore size distribution curve, it is believed that most of the peaks are from mercury crushing the aerogel. There is a regular peak located at 26 nm, indicating the existence of mesopores in TME. Because of the large pore sizes, aerogels would suffer modest capillary force during the drying process, it is possible to dry the aerogels at ambient pressure with proper solvents.

The aerogels can be well applied in oil absorption for their large porosity, the oil absorption capacity was measured in the same method as the previous work,^38^ In the study, six common organic solvents were taken to test the absorption of the four aerogels and the result was shown in Fig. S9.† TMM has the largest mass gain (7.9 to 20.9) for the organic solvents due to its largest porosity, exhibiting a high absorption capacity. And for each aerogel, the mass gain is larger for the organic solvents with larger density, which is coincident with the results of the previous reports.^11,38^

### Mechanical properties

**Fig. 7:**
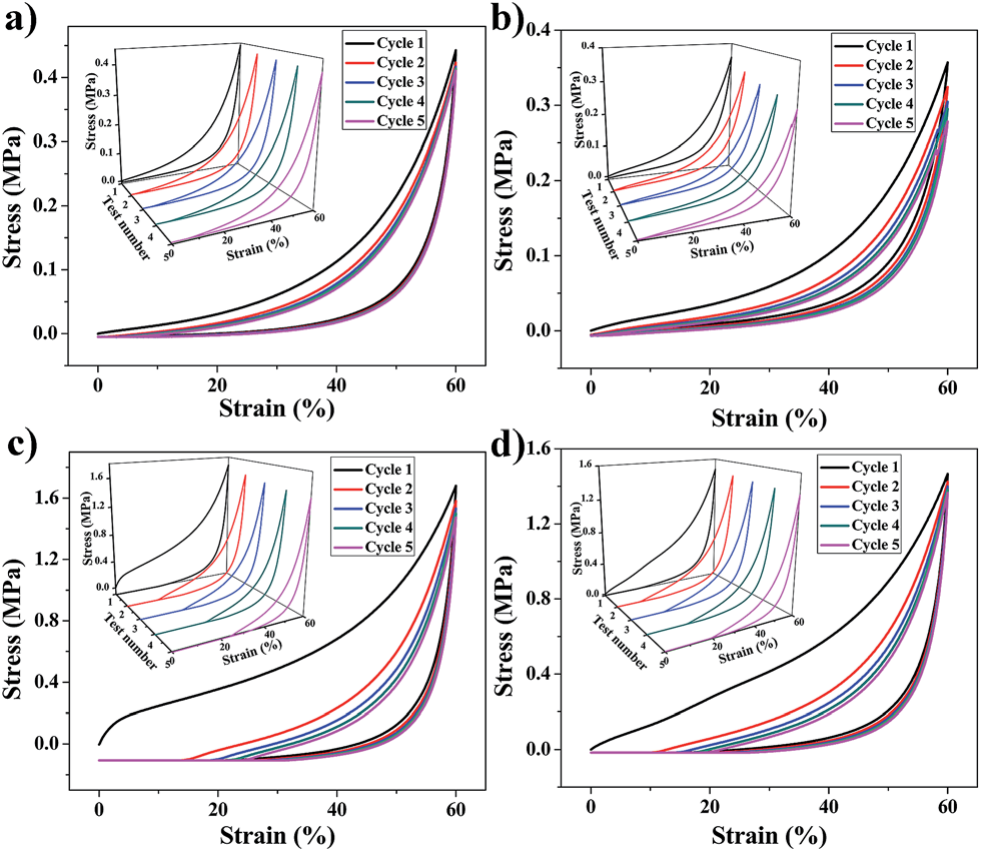
The stress–strain curves of the aerogels at repeating compression test, (a) TME, (b) TMM, (c) DME and (d) DMM.

**Fig. 8:**
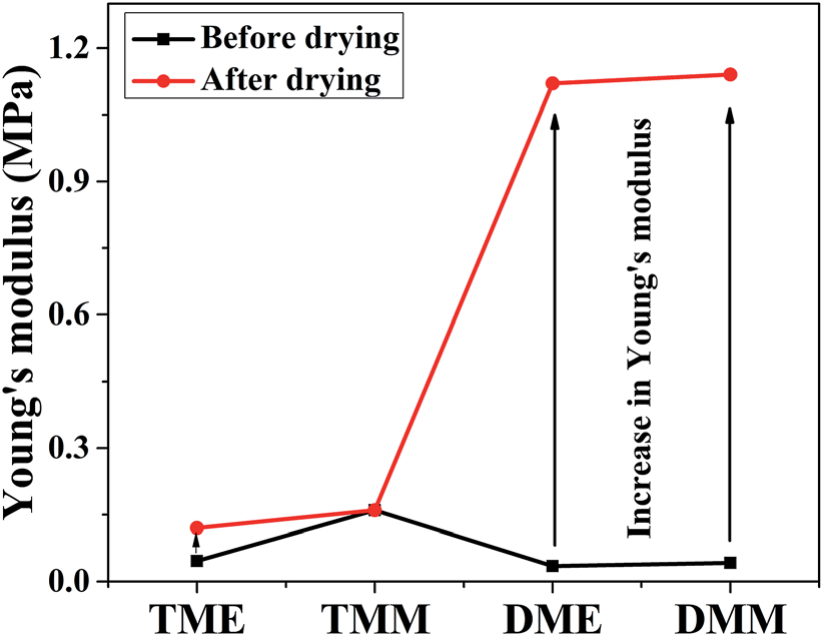
The Young's moduli of the gels before and after drying.

**Scheme 4:**
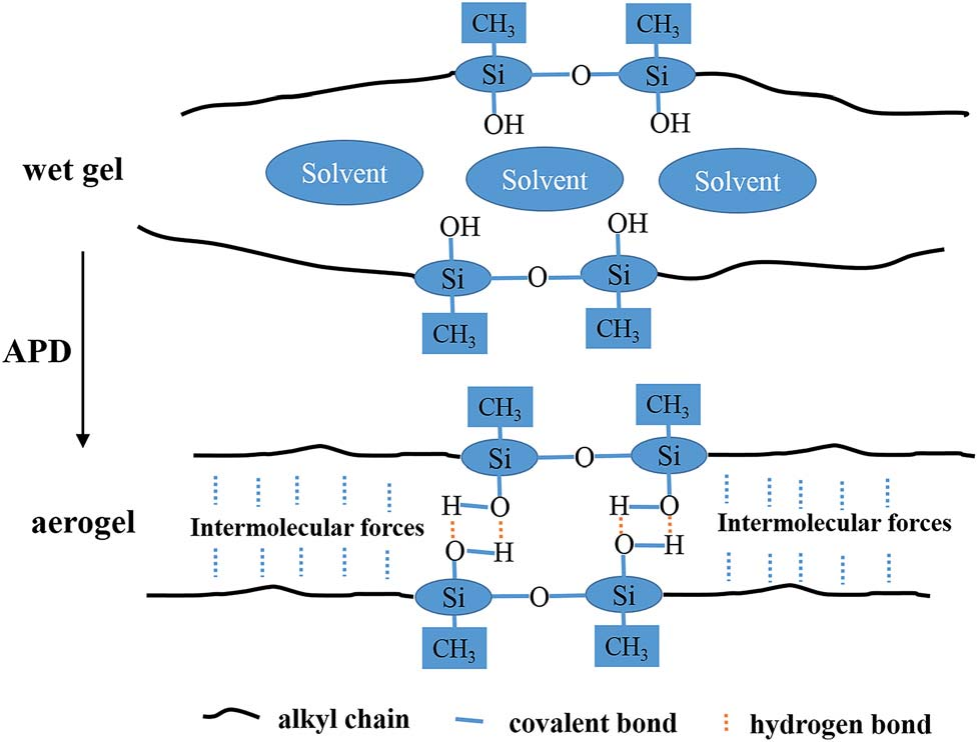
The illustration of the microstructures of the BMSQ aerogels before and after drying.

The mechanical properties of aerogels were tested using the 5-times-repeating uniaxial compression tests. As shown in Fig. 7, it takes more stress for the BMSQ aerogels to reach the same strain as BSQ aerogels because of the thicker networks of BMSQ aerogels. The Young's moduli of all the aerogels calculated from the initial slope of the stress–strain curves of the first compression are listed in Table 1, it is obvious that the Young's moduli of BMSQ aerogels are much larger, which means the BMSQ aerogels are more capable of resisting deformations. All the aerogels can withstand the deformation of 60% in the first cycle and show the obvious viscoelastic behaviors. TME shows relatively stabilized mechanical performance, there is not much difference between the stress–strain curves since the first cycle. TMM has obvious decreases in the Young's modulus and strength after each cycle and there are even structural fractures since the third cycle, which can be proved by the drops in the stress–strain curves at 58% deformation. Compared with TME, the poor mechanical property of TMM probably comes from the loosely assembled particles in the network. The stress–strain curves of DME and DMM resemble each other, they both show plastic deformations at compression and the decreases in strength and Young's modulus after each cycle. This is due to the similar composition and microstructure of the two aerogels. Fig. S10† shows the mechanical properties of the wet gels at repeating compression, it is interesting to find that the Young's moduli of DME and DMM increase dramatically after drying. The Young's moduli of the wet gels TME, TMM, DME and DMM are calculated to be 0.046, 0.163, 0.035 and 0.042 MPa respectively. Fig. 8 shows the Young's moduli of the gels before and after drying. Except the shrinkage, the possible reason for the increase of the Young's moduli of DME and DMM is presented as Scheme 4. For the low cross-link density and long linear segments in the BMSQ aerogels, the small size solvent molecules can permeate into the linear segments. After drying, the solvent molecules disappear, the intermolecular forces between the linear segments increase and the hydrogen bonds between the hydroxyl groups is generated, so the dried BMSQ aerogels are more capable to resist deformation.

Although the products were prepared from a very simple procedure, they exhibit good mechanical performance. A comparison of the mechanical properties between TME, DME and the flexible aerogels from the previous works is listed in Table 2, showing the good properties of the products.

**Table 2:** The comparison of the mechanical properties of the flexible aerogels^a^

Aerogels	Drying method	*ρ* (g cm^−3^)	*ε*_max_ (%)	*E* (MPa)	Ref.
BSQ aerogels	SCD	0.248	40	5.9	39
PVPMS aerogels	SCD	0.19	80	3.3	40
MTMS aerogels	SCD	0.05	50	0.0047	41
MTES-PDMS aerogels	APD	0.064	60	0.03	42
TME	APD	0.15	60	0.12	This work
DME	APD	0.17	60	1.12	This work

a *ρ*: density; *ε*_max_: the maximal deformation of aerogel; *E*: Young's modulus; SCD: supercritical drying.

## Conclusions

Aerogels with large pore size and robust skeleton were successfully dried at ambient pressure using methanol or ethanol as solvent without additional solvent exchange. The shrinkage of aerogels is increased when solvent with higher boiling point is used. The BMSQ aerogels are hydrophobic and the BSQ aerogels are hydrophilic due to the more methyl groups of the BMSQ aerogels. The particle sizes of the BMSQ aerogels are much larger and the networks are thicker due to their low reaction rate in the sol–gel process. The specific surface areas of the BMSQ aerogels are smaller than that of the BSQ aerogels. The BMSQ aerogels show plasticity as well as viscoelasticity in the compression test while the BSQ aerogels show viscoelasticity only. The long linear segments in the BMSQ aerogels endow them with different mechanical behaviours in compression before and after drying. The aerogels prepared using ethanol or methanol as solvent show the differences in the structure and properties. Aerogels gel much slower in ethanol. The solvents have much more influences on the BSQ aerogels than BMSQ aerogels. BSQ aerogels prepared with ethanol solvent have larger specific surface areas, smaller particle sizes in the networks and better mechanical performance in the compression test.

## Conflicts of interest

There are no conflicts to declare.

## Supplementary Material

RA-009-C8RA08646J-s001
