# Supplementary material for: Directly ambient pressure dried robust bridged silsesquioxane and methylsiloxane aerogels: effects of precursors and solvents
Source: RSC Adv. 2019 Mar 15;9(15):8664–71. doi: 10.1039/c8ra08646j (PMC9061811; doi:10.1039/c8ra08646j)
Supplement: RA-009-C8RA08646J-s001 [file RA-009-C8RA08646J-s001.pdf]

Electronic Supplementary Material (ESI) for RSC Advances.
This journal is © The Royal Society of Chemistry 2019

Supporting Information

Directly ambient pressure dried robust bridged silsesquioxane and methlysilxoane aerogels: Effects of precursors and solvents

Dangjia Chen, Hongyi Gao, Panpan Liu, Pei Huang and Xiubing Huang*

Beijing Advanced Innovation Center for Materials Genome Engineering, Beijing Key Laboratory of Function Materials for Molecule & Structure Construction, School of Materials Science and Engineering, University of Science and Technology Beijing, Beijing 100083, PR China.

*Corresponding author at: No. 30, Xueyuan Road, Haidian District, Beijing City, PR China.

*E-mail: hygao2009@163.com (H. Y. Gao); Tel.: +86-10-62333765.

Table S1. The detailed formulas of the products.

Sample	APTES (mmol)	APDEMS (mmol)	MPA (mmol)	Ethanol (mL)	Methanol (mL)	Water (mL)
TME	4.27	0	2.13	7	0	0.5
TMM	4.27	0	2.13	0	7	0.5
DME	0	4.27	2.13	7	0	0.5
DMM	0	4.27	2.13	0	7	0.5

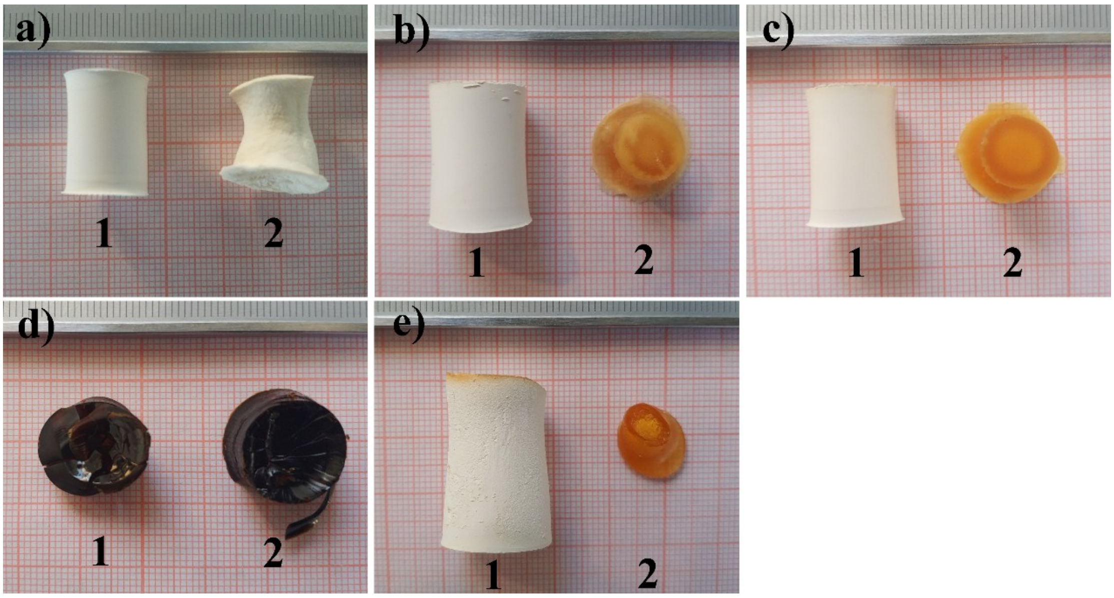

Figure S1. The optical images of aerogels prepared from different precursors and solvents; aerogels prepared from BSQ precursor are marked with 1, from BMSQ precursor are marked with 2, a) propanol, b) isopropanol, c) n-butyl alcohol, d) acetone and e) N,N-dimethylformamide are used as solvents; the shrinkage is calculated to be about a1) 34.1%, b1) 23.2%, c1) 34.7%, e1) 13.9%; gels prepared from BMSQ precursor are badly shrunken and twisted after drying; gels prepared using acetone as solvent are deep red translucent monoliths and they fragmented into pieces after drying.

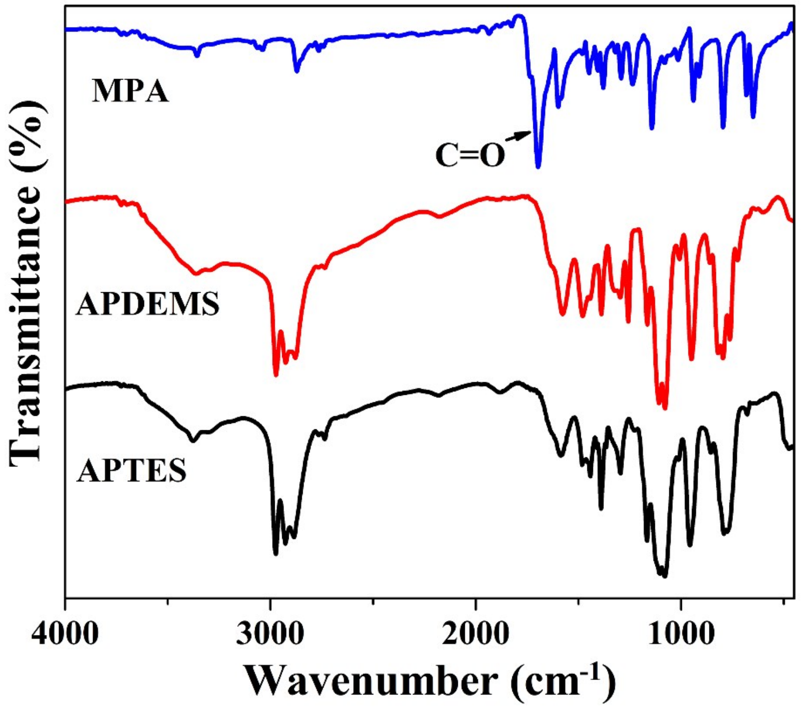

Figure S2. The FT-IR spectra of the start monomers of the aerogels.

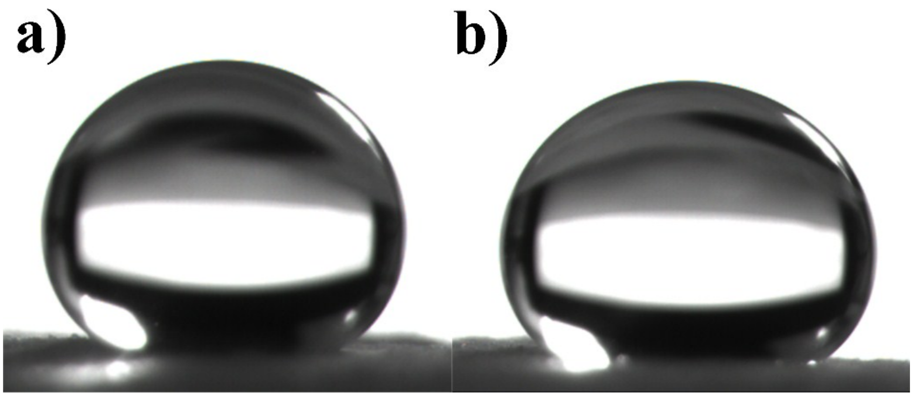

Figure S3. Water droplet on the surface of a) DME, b) DMM.

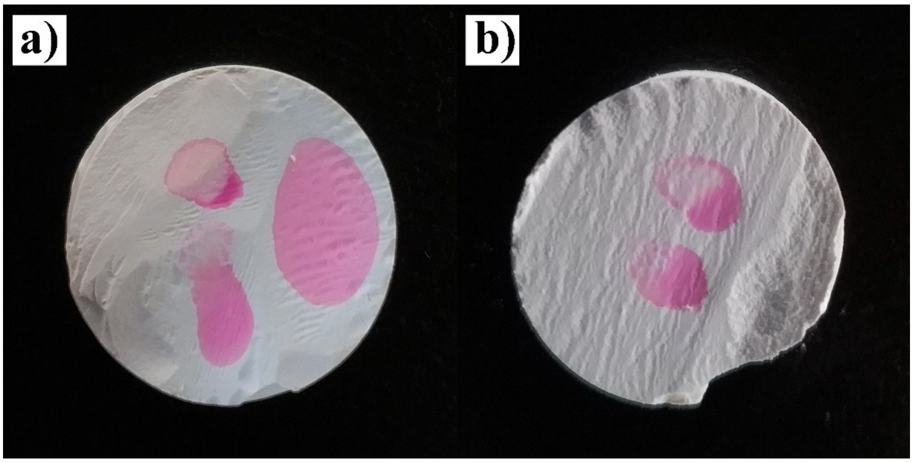

Figure S4. Water dyed with rhodamine B absorbed by a) TME, b) TMM.

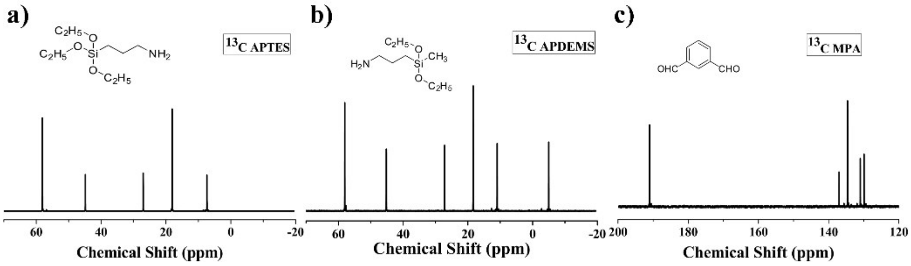

Figure S5. The ^{13}C NMR spectra of the start monomers.

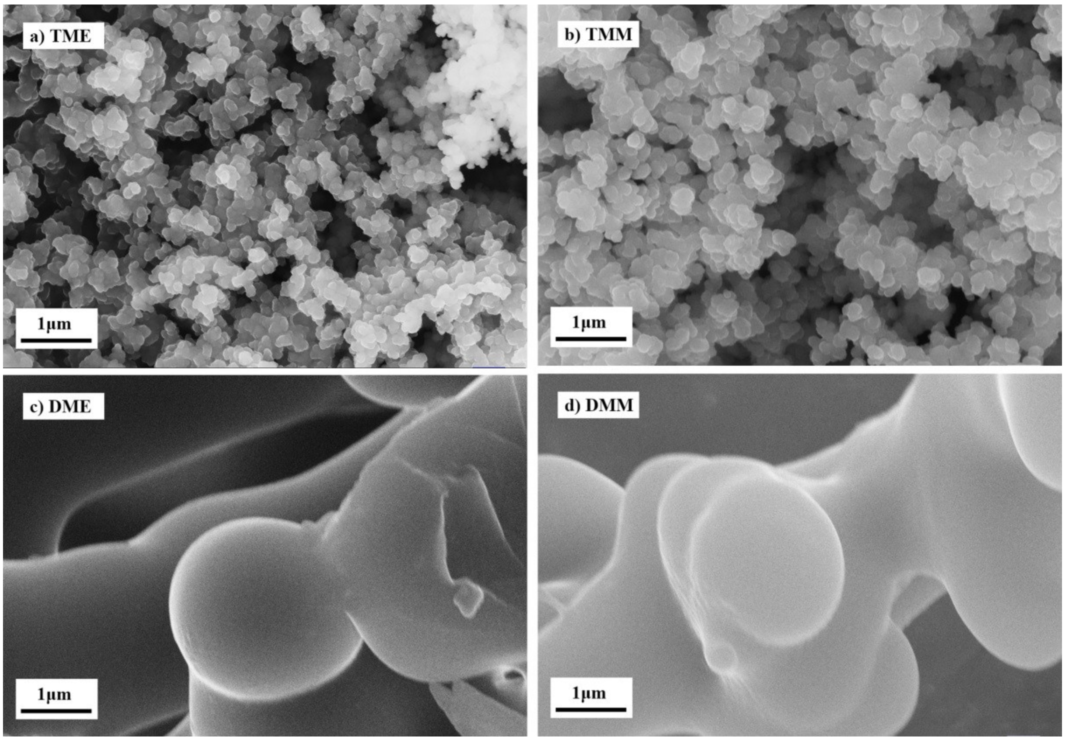

Figure S6. The SEM images of the aerogels with 40000 times magnification.

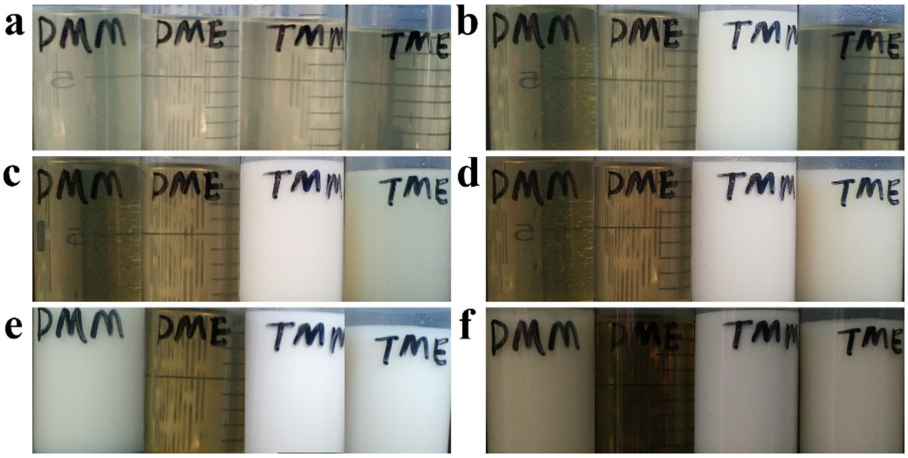

Figure S7. Photos of the sol-gel process taken every 2 hours, showing the reaction of different samples in the sol-gel process.

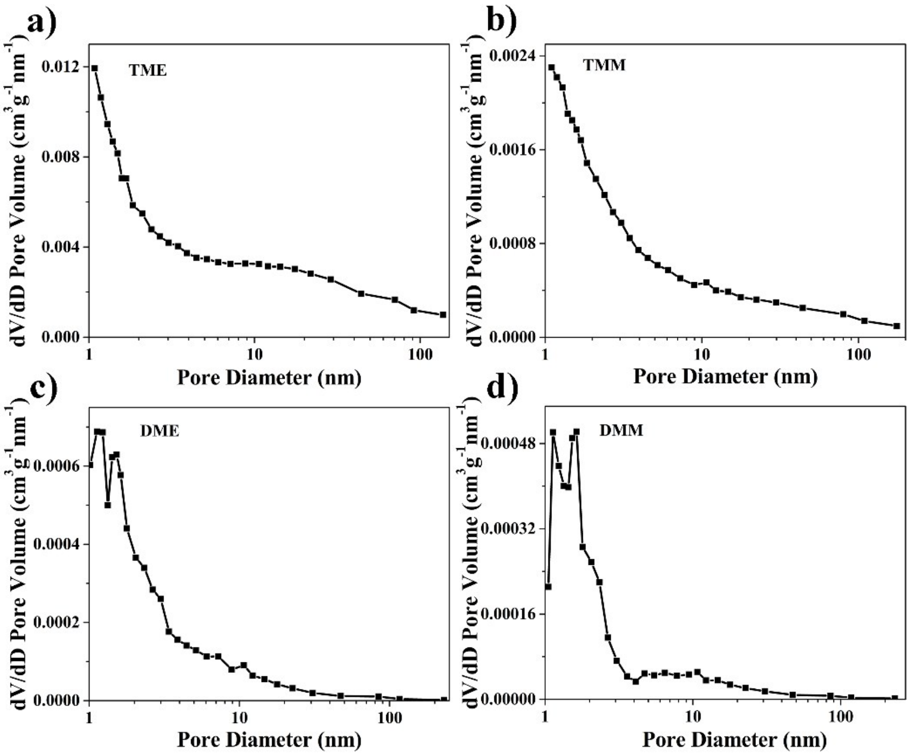

Figure S8. The pore size distribution of the aerogels.

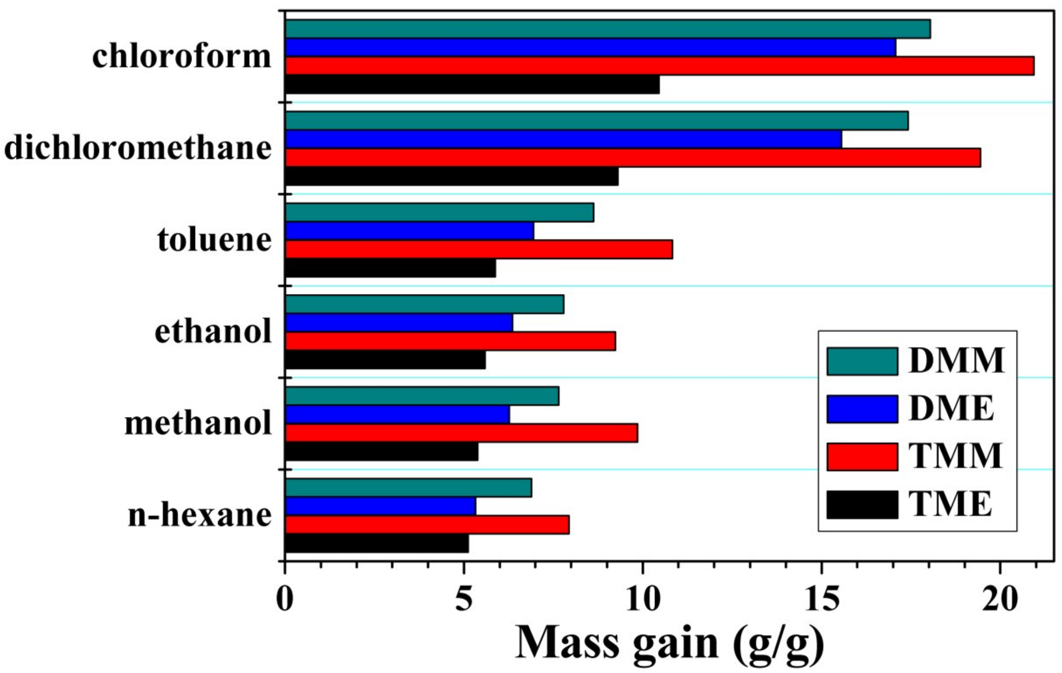

Figure S9. The oil absorption capacity of the aerogels.

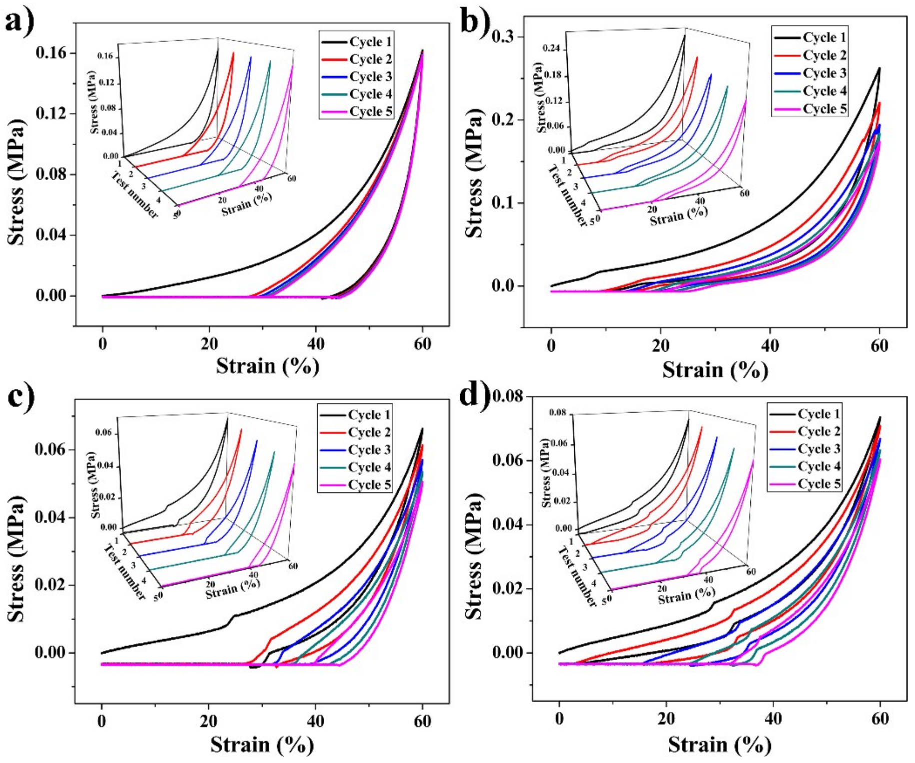

Figure S10. The stress-strain curves of the undried gels, a) wet TME, b) wet TMM, c) wet DME and d) wet DMM.
